# Hippocampal differential expression underlying the neuroprotective effect of delta-9-tetrahydrocannabinol microdose on old mice

**DOI:** 10.3389/fnins.2023.1182932

**Published:** 2023-07-18

**Authors:** Guy Shapira, Ifat Israel-Elgali, Meitar Grad, Eden Avnat, Lital Rachmany, Yosef Sarne, Noam Shomron

**Affiliations:** ^1^Faculty of Medicine, Tel Aviv University, Tel Aviv, Israel; ^2^Edmond J Safra Center for Bioinformatics, Tel Aviv University, Tel Aviv, Israel; ^3^Sagol School of Neuroscience, Tel-Aviv University, Tel Aviv, Israel

**Keywords:** aging, tetrahydrocannabinol, neurodegenerative diseases, neuroprotection, endocannabinoid system

## Abstract

Delta-9-tetrahydrocannabinol (THC) is the primary psychoactive compound of the cannabis plant and an exogenous ligand of the endocannabinoid system. In previous studies, we demonstrated that a single microdose of THC (0.002 mg/kg, 3–4 orders of magnitude lower than the standard dose for rodents) exerts distinct, long-term neuroprotection in model mice subjected to acute neurological insults. When administered to old, healthy mice, the THC microdose induced remarkable long-lasting (weeks) improvement in a wide range of cognitive functions, including significant morphological and biochemical brain alterations. To elucidate the mechanisms underlying these effects, we analyzed the gene expression of hippocampal samples from the model mice. Samples taken 5 days after THC treatment showed significant differential expression of genes associated with neurogenesis and brain development. In samples taken 5 weeks after treatment, the transcriptional signature was shifted to that of neuronal differentiation and survival. This study demonstrated the use of hippocampal transcriptome profiling in uncovering the molecular basis of the atypical, anti-aging effects of THC microdose treatment in old mice.

## Introduction

Components of the endocannabinoid system are increasingly studied for their potential as drug targets for neurodegenerative diseases, as well as psychiatric and metabolic conditions (Di Marzo et al., 2015; Friedman et al., 2019; Junior et al., 2020). Despite the recent progress, this system remains a challenging therapeutic target, due to the complex pharmacological profile of both its endogenous and exogenous ligands (Dainese et al., 2020; Fletcher-Jones et al., 2020).

In previous studies, we found that an acute microdose of the plant-derived cannabinoid ligand delta-9-tetrahydrocannabinol (THC) 0.0005–0.01 mg/kg (3–4 orders of magnitude lower than the standard dose for rodents. Any dose within this range is considered a microdose) elicits a distinct response that differs, and even partly contradicts the response to a standard dose of THC (Tselnicker et al., 2007). Administration of a THC microdose to healthy young (2 months old) mice elicited significant cognitive deficits, which lasted at least 5 months (Tselnicker et al., 2007).

In contrast to the above, the same THC microdose, administered shortly before or after an acute insult, protected mice from the resulting cognitive impairments. This protection was effective against a variety of acute insults, including neuroinflammation, epileptic seizures and exposure to various neurotoxins (Assaf et al., 2011; Fishbein et al., 2012; Fishbein-Kaminietsky et al., 2014).

In a follow-up study of old mice, a single THC microdose injection caused reversal of age-related cognitive decline, by inducing persistent biochemical and morphological brain alterations that lasted over 7 weeks after the treatment (Sarne et al., 2018). A wide variety of cognitive assays revealed that, 3–7 weeks after treatment, THC-treated old (24 months) mice significantly improved their performance, almost matching that of the untreated young mice. The effect was consistent across cognitive domains, including learning, avoidance and memory. Five weeks after THC administration, imaging results showed substantial morphological brain alterations in old treated mice. The tissue density of 11 brain regions significantly increased. In the entorhinal cortex, amygdala, external capsule-corpus callosum and visual cortex, tissue density increased by over 10%. No decreases in tissue density were detected. Additionally, the volume of four regions changed drastically in both brain hemispheres; most notable was a 21% reduction in amygdala volume and a 24% increase in entorhinal cortex volume (Sarne et al., 2018). Sirtuin 1, an enzyme with well-supported neuroprotective properties (Lee et al., 2019) showed significantly increased protein abundance, both in the prefrontal cortex and hippocampus of treated old mice. Although the above findings are highly indicative of an underlying neurological process, a cause-and-effect relationship between the biochemical, morphological and behavioral aspects is yet to be established.

To elucidate the mechanism underlying the anti-aging effects of the THC microdose, we compared the hippocampal gene expression profiles of the old mice (24 months old), 5 days and 5 weeks after THC microdose administration, with a matching, vehicle-treated control group.

We focus on the hippocampus primarily due to its significant MRI and biochemical essay results from Sarne et al. (2018) and its high density of CB1 receptors. The 5 days and 5 weeks post-treatment timepoints were chosen for coinciding with the appearance of neurogenic markers and the timing of the behavioral essays (Sarne et al., 2018), respectively.

## Materials and methods

### Animals and treatment

Experiments were performed on old (24 months old) female and young (2 months old) male mice of the Institute of Cancer Research. The animals were housed 5–10 per cage in the Animal Care Facility at a temperature of 21°C and a 14/10-h light/dark cycle, with free access to food and water. All the mice were treated as described by Sarne et al. (2018). Briefly, they were injected intraperitoneally with either THC:ethanol:cremophor:saline vehicle (THC dose of 0.002 mg/kg; THC donated by Prof. Mechoulam, the Hebrew University, Jerusalem) or a THC-free vehicle solution in a single administration, in a total volume of 0.1 mL/10 g bodyweight. RNA sequencing of the hippocampus was performed on 5 mice of each of the 5 groups (25 mice overall).

### RNA extraction and sequencing

Five weeks post treatment, the mice were sacrificed by cervical dislocation. The brains were removed and their hippocampus dissected. Hippocampus tissue samples underwent RNA purification using phenol-chloroform extraction, and were then sent to sequencing at Macrogen Inc., using the Illumina platform, following a TruSeq RNA library v2 preparation.

### Real-time polymerase chain reaction

Reverse transcription reactions for mRNA were performed using the High-Capacity cDNA Reverse-Transcription Kit with random primers, according to the manufacturer’s recommendations (Thermo Fisher Scientific, United States). Real-time polymerase chain reaction (RT-PCR) was performed to validate top significant candidates obtained by RNAseq analysis, according to the manufacturer’s protocol (Thermo Fisher Scientific, United States). Normalization for mRNA was performed compared to GAPDH expression.

### Bioinformatics analysis

Adaptors were trimmed using cutadapt (Martin, 2011), and transcripts were quantified using Salmon 0.8.1v (Patro et al., 2017) with the Gencode vM17 mouse assembly. The clustering of gene quantification data was assessed using variance stabilizing transformation and principal component analysis. A small subset of extreme outlier samples was removed from further analysis. The remaining samples were fitted to a negative binomial distribution, a generalized linear model for differential expression testing under the standard DESeq2 method (Love et al., 2014). Surrogate variable analysis (Leek et al., 2012) was used to correct for batch effect, according to sample preparation batches. Heatmaps were made with the ComplexHeatmap package (Gu et al., 2016). Heatmaps comparing differential expression used log2FoldChange values. Heatmaps comparing expression patterns used mean expression per group, with variance stabilization (Huber et al., 2002) and Z-scoring for visualization. Global gene enrichment analysis was performed using GOrilla (Eden et al., 2009), with complete lists of genes sorted by value of ps as input. This was done in order to overcome the possibility of relevant differentially expressed genes failing to pass the strict significance threshold. Heatmap creation and expression pattern clustering used genes with uncorrected value of ps lower than 0.001. Gene set enrichment was performed of individual and ranked gene-sets, and relevant publications were searched through text mining, using (STRING v11, n.d.).

False discovery rate correction of multiple comparisons was performed using the Benjamini and Hochberg approach (Benjamini and Hochberg, 1995). Significant differentially expressed genes were defined as protein coding genes, with mean normalized expression values greater than 5 and adjusted *p*-values of 0.05 or less. Genes that were not significant after multiple comparison correction, but had an uncorrected p-value of 0.005 or lower were defined as “significant before correction” and used for enrichment analysis and quantitative PCR verification.

## Results

From whole-hippocampus samples obtained from old female mice, 5 days and 5 weeks after THC microdose treatment, mRNA was extracted; and compared to mRNA of a vehicle treated group. Similarly, mRNA from young male mice, 5 weeks after THC microdose treatment, was compared to mRNA of a vehicle treated group. Significant differential expression was detected in all the comparisons, with the untreated mice of the same age serving as the reference group for each comparison. Due to their sexual difference, the young and old groups are never compared directly. In old mice, both 5 days and 5 weeks after treatment, 18 genes with significantly different expression were identified; none of them were common between the two groups. In young mice 5 weeks after treatment, 88 genes were identified with significantly different expression; none were identified as significant in any of the comparisons of old mice.

**Figure 1:**
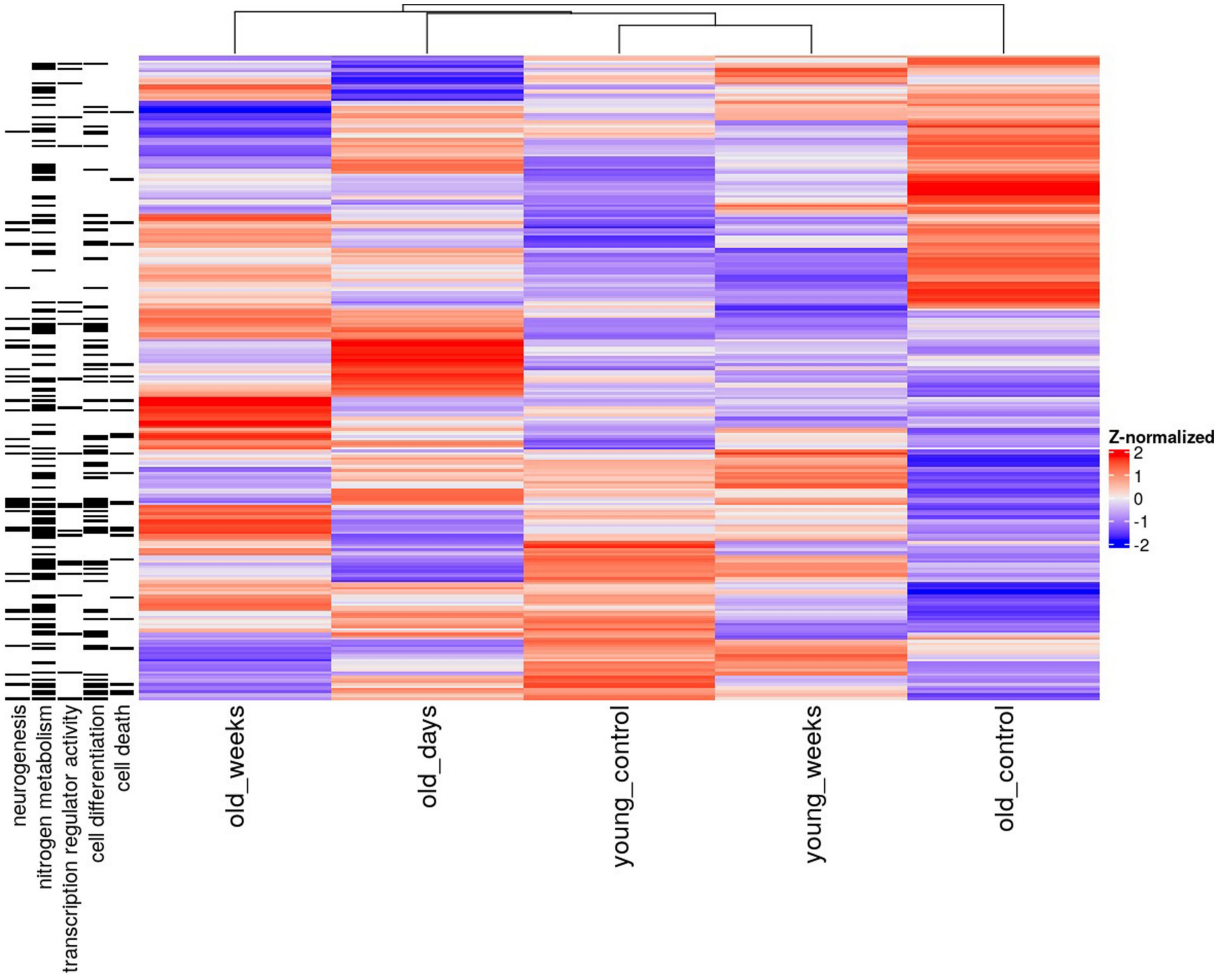
Heatmap of mean hippocampal gene expression of young and old mice 5 days or 5 weeks after treatment with THC or vehicle solution (control). Only genes with significant differential expression before correction (*p* < 0.005) in the old groups are shown. The annotations on the left denote associations with a gene ontology term.

**Figure 2:**
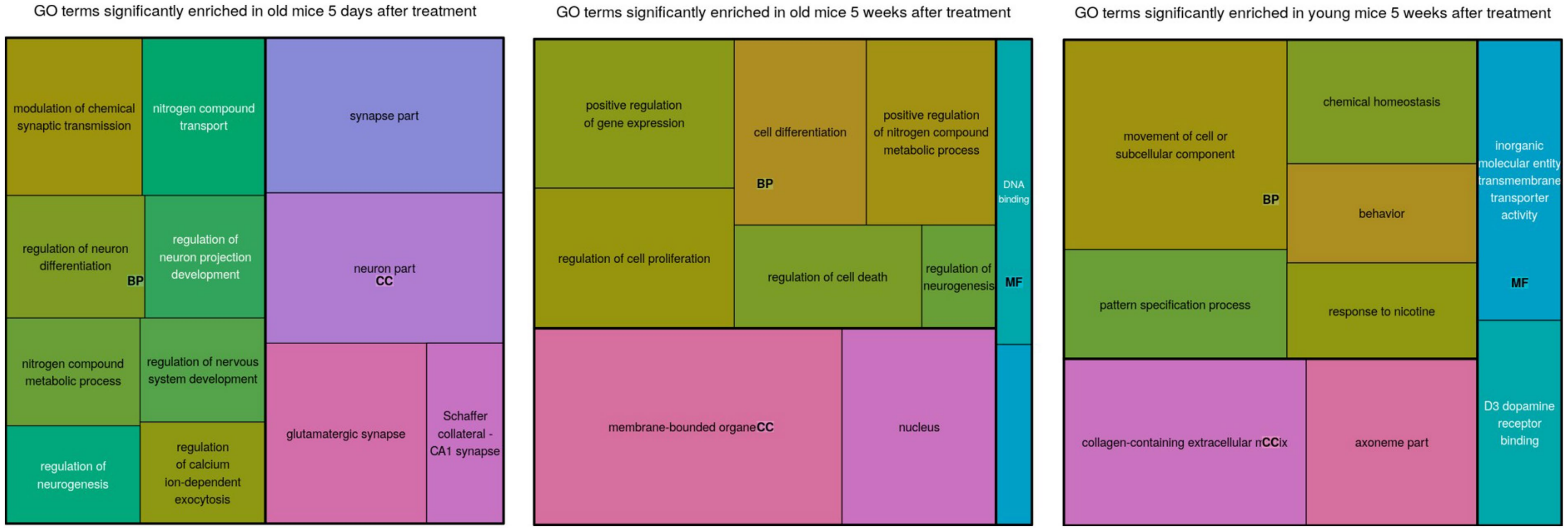
A treemap of enriched gene ontology (GO) terms for each comparison. The sizes of the blocks represent relative enrichment significance. The terms are clustered by ontology: BP, Biological Process; MF, Molecular Function; CC, Cellular Compartment.

The pattern of mutually exclusive difference in gene expression suggests a large degree of specificity, by which the same treatment elicited highly divergent effects depending on the age of the mouse models and the time elapsed since the treatment. These group-specific differences were further supported by the functional enrichment results, thus suggesting that distinct differentially expressed genes are also functionally distinct (Figures 1, 2).

Differentially expressed genes in old mice, both days and weeks after treatment, were enriched in functional groups of neurogenesis, nitrogen metabolism, transcription factor activity, cell differentiation and cell death. The old mice were clustered closer to the young mice, after treatment compared to before treatment. This is consistent with the cognitive assay results [see the Discussion in Sarne et al. (2018); Figure 2].

Genes involved in the regulation of neurogenesis were highly enriched in the old group that was 5 days after treatment, and much less enriched in the other groups. Notably, the neurogenesis biomarker Doublecortin (DCX) was also detected around the same time-frame (Sarne, 2019). This supports the idea that neurogenesis coincided with differential expression of known neurogenic genes. Genes associated with nitrogen metabolism were also enriched among the old mice that were treated, but not among the young mice that were treated.

### Reversal of age-associated decline in the expression of pro-neurogenic genes in old mice, 5 days after THC microdose treatment

**Figure 3:**
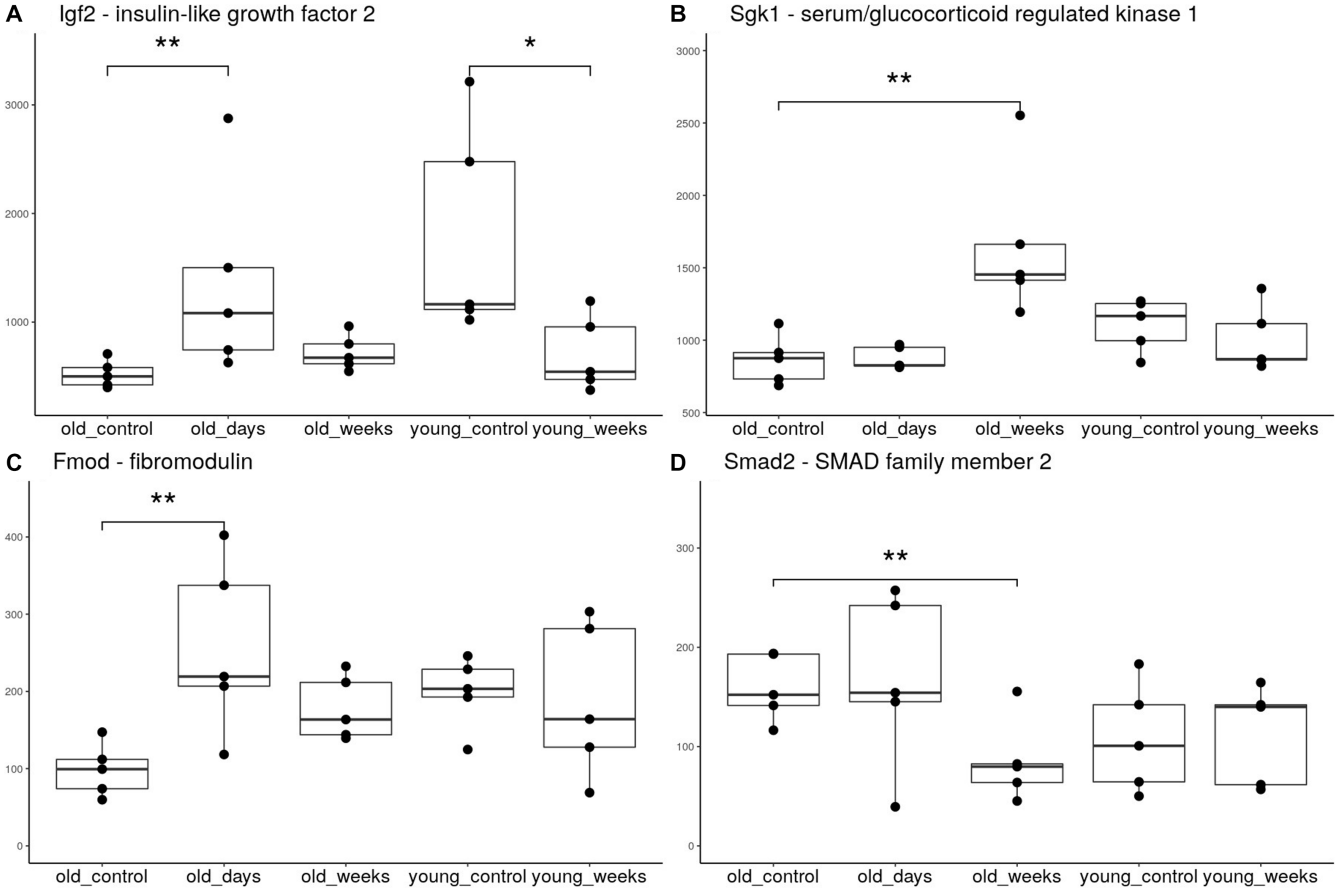
Boxplots of hippocampal expression of selected genes. The groups included young and old mice 5 days or 5 weeks after treatment with THC or vehicle solution (control). Each group consists of 5 mice. **p < 0.05 significance indicators denote significant differential expression after applying the false discovery rate correction, *p < 0.05 significant differential expression before correction.

The THC treatment reversed the age-associated trend in the expression of numerous genes at the 5-day timepoint. The hippocampal expression of Igf2 in old mice nearly doubled 5 days after the THC treatment (Figure 3A). The young group showed an opposite trend, by which Igf2 expression decreased 5 weeks after treatment; this was verified by RT-PCR.

Fibromodulin (Fmod) is another gene with age-dependent down-regulation (Roughley et al., 1996) that was upregulated in the old group days after THC treatment (Figure 3B). The expression of the collagen genes, Col1a2 and Col1a1, known to be regulated by Olsson et al. (2017), and directly interact with Fmod (Kalamajski et al., 2016), also exhibited the same upregulation. The thyroid hormone receptor β gene (Thrb) is an additional gene with age-dependent decline in expression (Pawlik-Pachucka et al., 2018), which was significantly upregulated as a result of THC treatment.

The aforementioned genes, which all demonstrated age-associated downregulation that was reversed by the THC treatment, were previously shown to be associated with adult neurogenesis, either stable or induced, as a part of a recovery mechanism (Kakoi et al., 2012; Sohur et al., 2012; Ferrón et al., 2015; Liu and Brent, 2018). Other neurogenic genes that are upregulated at this timepoint include Cdh1, which supports neuronal differentiation and survival with its cofactor APC/C (Delgado-Esteban et al., 2013), and Zic2, a vital transcription factor for early brain development and migration of neuronal populations (McMahon and Merzdorf, 2010; Murillo et al., 2015). The timing of the differential expression of these genes is also significant, as it coincides with the protein expression of the neurogenesis biomarker DCX, in the hippocampus of old treated mice, at the same 5-day timepoint (Sarne, 2019).

### Transcriptional signature of neuronal differentiation, survival and neuroprotection in old mice, 5 weeks after THC microdose treatment

Five weeks after the THC treatment, the pattern of differential expression was very distinct from that at the earlier 5-day timepoint, but shared some functional similarities. Sgk1, a major transcription regulator that is essential for activation of the neurogenic hedgehog pathway (Anacker et al., 2013), was significantly upregulated 5 weeks after treatment (Figure 3C). Smad2, an inhibitor of TGF-β mediated neurogenesis and axonal morphogenesis (Stegmüller et al., 2008; Míguez et al., 2013), was significantly downregulated (Figure 3D). This suggests disinhibition of these neurogenic pathways. The transcription factor Neurod2, which is required for neuronal differentiation and later neurodevelopment (Wilke et al., 2012; Bayam et al., 2015), was also upregulated at the 5-week timepoint.

In contrast to the above, a more common and significantly enriched function of the THC treatment was that of neuroprotection and cell survival. NeuroD2, Mc1r, Timp3 and Nfkbia were all upregulated at 5 weeks after treatment, and were associated with protection against neuroinflammation, promotion of cell survival, and attenuation of neuronal and cognitive damage in numerous animal models (Fridmacher et al., 2003; Gibb et al., 2015; Fu et al., 2020; Pang and Shi, 2021; Xu et al., 2021). Among these upregulated genes, Sgk1 in particular was associated with cell survival in the aging brain (Sahin et al., 2013), and highly protective against a broad range of neurotoxins (Iqbal et al., 2015) and pathologies (Lee et al., 2020; Martin-Batista et al., 2021).

## Discussion

The extraordinary, long-lasting effect of a single microdose of THC on old rodent brains requires thorough investigation for underlying mechanisms of action. We found distinct transcriptional signatures of gene expression in the hippocampus of these model animals, at different times after treatment. Many of the differentially expressed genes were associated with a broad range of effects beneficial to the aging brain, including neurogenesis, neuroprotection and anti-neuroinflammation.

The significant hippocampal upregulation of Igf2, at 5 days after THC treatment, is strongly implicated in many of the beneficial anti-aging effects that were observed in the cognitive assays. Administration of Igf2 peptides to the hippocampus of old rats was shown to alleviate much of their age-associated memory deficits (Steinmetz et al., 2016); the role of Igf2 in memory enhancement corroborates prior studies (Beletskiy et al., 2021). In addition to its cognition enhancing effect, Igf2 has been studied for its potential as a neuroprotective agent (Beletskiy et al., 2021). This strongly implies its possible involvement in the broad neuroprotective effect of THC microdose treatment, as described in our previous research (Sarne et al., 2011; Fishbein-Kaminietsky et al., 2014). The extent to which Igf2 upregulation is required for these effects is yet to be determined. The downregulation of Igf2 in the young treated group might be implicated in their observed cognitive decline (Sarne et al., 2018), contrasting the beneficial treatment effect seen in old mice, regardless of sex (Tselnicker et al., 2007; Sarne et al., 2018).

While neurogenesis persists to some degree in a few, small neurogenic niches, it greatly diminishes with age and does not compensate for the gradual death of neurons. The significant increase in posterior hippocampal volume (by about 13% according to the T2 MRI) (Sarne et al., 2018) and the marked improvement in cognitive functions associated with this brain region are clear outliers of the norm for an aging brain. Several of the differentially expressed genes were associated with neurogenesis, especially at the 5-day timepoint. This coincides with the neurogenic molecular markers. Another factor that may have contributed to this phenomenon is the upregulation of genes associated with cell survival, which was prominent 5 weeks after the treatment. Missing from the differential expression results were two genes that were previously hypothesized to be involved in anti-aging effects, Sirtuin-1 (Sirt1) and brain-derived neurotrophic factor (Bdnf). Bdnf, a neurotrophic factor that is similarly associated with neuroprotection and brain aging (Molinari et al., 2020), was also found to have elevated protein expression levels in the hippocampus and pre-frontal cortex of young mice (8 weeks old). Seven weeks after the same THC treatment, hippocampal mRNA levels of Bdnf were unchanged. Sirt1 is a well-studied neuroprotective factor that has been shown to improve the overall neurological condition in aging and neurodegenerative disorders. Sirt1 expression was shown to decrease with age (Lee et al., 2019). Sarne et al. evaluated Sirt1 protein expression level in both the hippocampus and pre-frontal cortex of old mice 7 weeks after treatment (Sarne et al., 2018). However, our results show unchanged mRNA levels of Sirt1, both days and weeks after treatment. The protein expression levels of Sirt1 were shown to differ significantly from the mRNA expression levels (Rahman and Islam, 2011; Sarne, 2019; Molinari et al., 2020). Thus, the contradictory results are rather benign.

Our findings imply that the THC microdose treatment alleviates age-dependent cognitive deficits by modulating multiple hallmarks of brain aging, supporting past hypotheses regarding the relation between aging and the endocannabinoid system (Bilkei-Gorzo, 2012). Neuroinflammation, a major culprit of neurodegeneration and brain aging (Wang et al., 2022), is inhibited by a significant proportion of genes upregulated by the THC treatment. Sirt1 (Jiao and Gong, 2020), Igf2 (Guo et al., 2023), and Sgk (Han et al., 2022) are all independently associated with an anti-inflammatory response, but it is unclear if the observed effects depend on upregulation of any of these genes, alone or in combination. The same applies to the upregulated neuroprotective genes, such as Cdh1 (Li et al., 2017), Smad2 (Liu et al., 2015), Col1a1 and Col1a2 (Zheng et al., 2021), each individually proven to attenuate neuronal apoptosis. We hypothesize that the beneficial effect of the THC microdose is mediated by multiple differentially expressed genes, conferring a potentially synergistic effect by attenuating a multitude of age-related brain deficits.

In conclusion, differential expression patterns of genes were found to be associated with aging, neurogenesis and cognitive ability, in large agreement with the phenotypic findings. This provides a compelling, albeit incomplete hypothesis for the mechanisms driving the unusual effects of the THC microdose treatment on old mice. Follow-up studies are required to assess the involvement of individual genes and the potential therapeutic application of THC microdose treatment.

## Data availability statement

Full sequencing data will be shared upon reasonable request.

## Author contributions

LR and YS were responsible for animal handling. GS performed the RNA-seq data analysis and prepared the tables and figures. II-E and MG performed data acquisition and validation. GS, EA, and NS were responsible for writing the manuscript. All authors took part in conceiving the study, contributed to the article, and approved the submitted version.

## Conflict of interest

The authors declare that the research was conducted in the absence of any commercial or financial relationships that could be construed as a potential conflict of interest.

## Publisher’s note

All claims expressed in this article are solely those of the authors and do not necessarily represent those of their affiliated organizations, or those of the publisher, the editors and the reviewers. Any product that may be evaluated in this article, or claim that may be made by its manufacturer, is not guaranteed or endorsed by the publisher.
